# Internal jugular pressure increases during parabolic flight

**DOI:** 10.14814/phy2.13068

**Published:** 2016-12-30

**Authors:** David S. Martin, Stuart M. C. Lee, Timothy P. Matz, Christian M. Westby, Jessica M. Scott, Michael B. Stenger, Steven H. Platts

**Affiliations:** ^1^ KBRwyle Science, Technology & Engineering GroupHoustonTexas; ^2^ MEI TechnologiesHoustonTexas; ^3^ Universities Space Research AssociationHoustonTexas; ^4^ NASA Johnson Space CenterHoustonTexas

**Keywords:** Spaceflight, visual impairment and intracranial pressure

## Abstract

One hypothesized contributor to vision changes experienced by >75% of International Space Station astronauts is elevated intracranial pressure (ICP). While no definitive data yet exist, elevated ICP might be secondary to the microgravity‐induced cephalad fluid shift, resulting in venous congestion (overfilling and distension) and inhibition of cerebrospinal and lymphatic fluid drainage from the skull. The objective of this study was to measure internal jugular venous pressure (IJVP) during normo‐ and hypo‐gravity as an index of venous congestion. IJVP was measured noninvasively using compression sonography at rest during end‐expiration in 11 normal, healthy subjects (3 M, 8 F) during normal gravity (1G; supine) and weightlessness (0G; seated) produced by parabolic flight. IJVP also was measured in two subjects during parabolas approximating Lunar (1/6G) and Martian gravity (1/3G). Finally, IJVP was measured during increased intrathoracic pressure produced using controlled Valsalva maneuvers. IJVP was higher in 0G than 1G (23.9 ± 5.6 vs. 9.9 ± 5.1 mmHg, mean ± SD *P* < 0.001) in all subjects, and IJVP increased as gravity levels decreased in two subjects. Finally, IJVP was greater in 0G than 1G at all expiration pressures (*P* < 0.01). Taken together, these data suggest that IJVP is elevated during acute exposure to reduced gravity and may be elevated further by conditions that increase intrathoracic pressure, a strong modulator of central venous pressure and IJVP. However, whether elevated IJVP, and perhaps consequent venous congestion, observed during acute microgravity exposure contribute to vision changes during long‐duration spaceflight is yet to be determined.

## Introduction

Visual impairment, structural changes of the eye (optic disk edema, globe flattening, and choroidal folds), and optic nerve alterations (sheath dilatation, tortuosity, and kinking) have been documented in some astronauts completing long‐duration spaceflight on the International Space Station (ISS). While originally reported to affect ~50% of astronauts (Mader et al. 2011), more recent data suggest that the incidence may be as high as 75% and that men are affected to a greater degree than women (Barr et al. 2014). Understanding factors contributing to vision and ocular structure changes observed in astronauts who participate in long‐duration spaceflight has become a high priority for NASA, particularly because it has been identified as a potential health issue that could impact the success of exploration missions as well as the long‐term health of the individual astronaut. One hypothesized cause of these vision changes is increased intracranial pressure (ICP), leading to naming the condition the visual impairment and intracranial pressure (VIIP) syndrome (Michael and Marshall‐Bowman 2015). While an elevated ICP may arise from several etiologies, a leading hypothesis is that the cephalad fluid shift in weightlessness inhibits venous drainage, which can impair cerebrospinal and lymphatic fluid drainage from the skull, as described in the Monro‐Kellie hypothesis (Macintyre 2013). An additional source of increased ICP could be an elevated central venous pressure (CVP), which also might inhibit venous drainage, although previous data suggest that CVP is either unchanged or decreased during the first 1–2 day of spaceflight, as reported in four astronauts (Buckey et al. 1996; Foldager et al. 1996). However, no CVP data are available after several months of space flight when VIIP symptoms manifest.

Given that the internal jugular vein is a primary pathway for cerebral venous drainage, and the potential impact of venous drainage on the VIIP syndrome (Michael and Marshall‐Bowman 2015), we measured internal jugular vein pressure (IJVP) in normal gravity and in hypogravity during parabolic flight using compression sonography, a novel method used to noninvasively measure venous pressure. In this technique, a bladder filled with an ultrasound‐translucent mixture of water and glycerin and connected to a manometer is attached to the head of a linear array ultrasound probe. The ultrasound probe/bladder is then slowly pressed against the skin overlying a vein of interest until the vein is compressed to the point of closure as visualized on the ultrasound screen. In accordance with Laplace's law, the pressure which is applied to compress the vein is assumed to equal the pressure within the vein. The technique is similar in principal to indirect, auscultative blood pressure measurements in which systolic arterial pressure is equivalent to cuff pressure applied to the tissue overlying the brachial artery corresponding to the detection of the first Korotkoff sound, produced by the intermittent and turbulent rush of blood under the cuff at the opening of the artery as cuff pressure decreases. Previous work in our laboratory (Martin et al. 2015) and others (Thalhammer et al. 2007) examining compression sonography has confirmed the linear relation between the externally‐applied probe pressure and invasive measurements of peripheral vein pressure. More specifically, pilot data from our laboratory measured from +20° (head‐up) to −20° (head‐down) whole‐body tilt show a strong correlation within subjects (*n* = 2) between IJVP measured noninvasively using this compression sonography technique and an invasive measure with a pressure‐tipped transducer placed in the internal jugular vein (*R* = 0.87, 0.97; S.H. Platts, personal communication).

Thus, in this experiment we first sought to determine whether IJVP, as a potential contributor to elevated ICP, was increased from normal (1G) to weightlessness (0G). We hypothesized, based upon the venous distension commonly observed in space and parabolic flight (Arbeille et al. 2001), that IJVP would be higher during 0G than in 1G. In two subjects, we also sought to determine whether there was a graded effect of gravity level on IJVP. Second, we sought to determine whether IJVP would be elevated by controlled Valsalva maneuvers in 0G, which previously have been shown to increase venous pressure in 1G. Resistive exercise, routinely performed by ISS astronauts as a countermeasure to musculoskeletal deconditioning (Moore et al. 2010; Loehr et al. 2011) also has been suggested to be a contributing factor to VIIP, perhaps exacerbating venous overfilling (or congestion) through the performance of Valsalva maneuvers during daily exercise (Haykowsky et al. 2003). We hypothesized that controlled Valsalva maneuvers during parabolic flight, as a simulation of respiratory maneuvers possibly performed during resistive exercise countermeasures on ISS, would further elevate IJVP above the levels experienced with weightlessness alone.

## Methods

### Subjects

Eleven healthy volunteers (three males, eight females; age, mean: 39.5 years, range 27–60 yrs) participated in testing before and during parabolic flight (height, mean: 168 cm, range: 157–196 cm; weight, mean: 66.6 kg, range: 50.5–109.9 kg). All subjects completed a modified Air Force class III physical exam before participation. Subjects were asked to abstain from nicotine and alcohol for 12 h and high fat foods for 4 h prior to flight. Subjects were provided written and oral explanations of the test protocols and risks involved in participation. This study was approved by the NASA Johnson Space Center Institutional Review Board, and written informed consent was obtained from each test subject prior to participation.

### Overall study design

IJVP was measured before and during parabolic flight at rest and during controlled breathing maneuvers designed to increase intrathoracic pressure and CVP. Preflight baseline measures were obtained within 3 h of the flight with the subjects in the supine posture to allow for adequate filling of the jugular vein. After baseline measurements, subjects exhaled through a tube connected to a calibrated pressure manometer (Differential Pressure Manometer Model HD750, Extech Instruments, Nashua, NH) to pressures of 10 and 20 mmHg while maintaining an open glottis to increase intrathoracic pressure. IJVP measurements were repeated at each pressure. All measurements were taken at the end of a quiet expiration, and a minimum of three pressure measurements were obtained for each condition. The location at which IJVP measures were obtained during the preflight baseline condition was marked on the neck to allow for uniform measurement location of IJVP measures across all conditions. Specifically, IJVP measures were obtained in a section of the vessel where the walls could be clearly visualized during compression, being as superior as possible while avoiding the confluence with the superior thyroid vein.

IJVP measures at rest and with breathing maneuvers were repeated during parabolic flight. Four flights were allotted for this experiment, with each subject flying only once and the corresponding preflight measures obtained on the same day. Each flight lasted approximately 2.5 h and included 40 parabolas, providing about 20 sec of 0G or partial gravity per parabola. Of the 40 parabolas, 32 achieved 0G, four approximated lunar gravity (0.17G), and four approximated Martina gravity (0.29G). Each reduced gravity parabola was preceded and followed by a hypergravity phase of up to 1.8G lasting about 20 sec. Inflight measures were obtained while the subjects were seated on the floor of the aircraft, with legs positioned horizontally. IJVP data were obtained after at least 5 sec into each parabola when the vein appeared to be fully distended. Because of the limited time available during the parabolic flight, breathing maneuvers were performed by only 7 of the 11 subjects. Additionally, while 1G and 0G resting measures were obtained in all subjects, IJVP during parabolas intended to simulate lunar and Martian gravity was measured in only two subjects (one male, one female) due to parabolic flight plan constraints not specific to this study. No breathing maneuvers were performed during partial gravity parabolas.

### Noninvasive internal jugular venous pressure

Noninvasive IJVP was measured using a custom‐made prototype compression sonography device (VeinPress 2010, VeinPress GmbH, Switzerland). This device has not been approved by the FDA for clinical use and has only been used for investigational purposes (Thalhammer et al. 2007, 2009; Uthoff et al. 2010). Use of the VeinPress in this experiment was approved by the NASA JSC IRB after review by NASA and NASA‐contractor safety experts and determined to be a nonsignificant risk.

**Figure 1:**
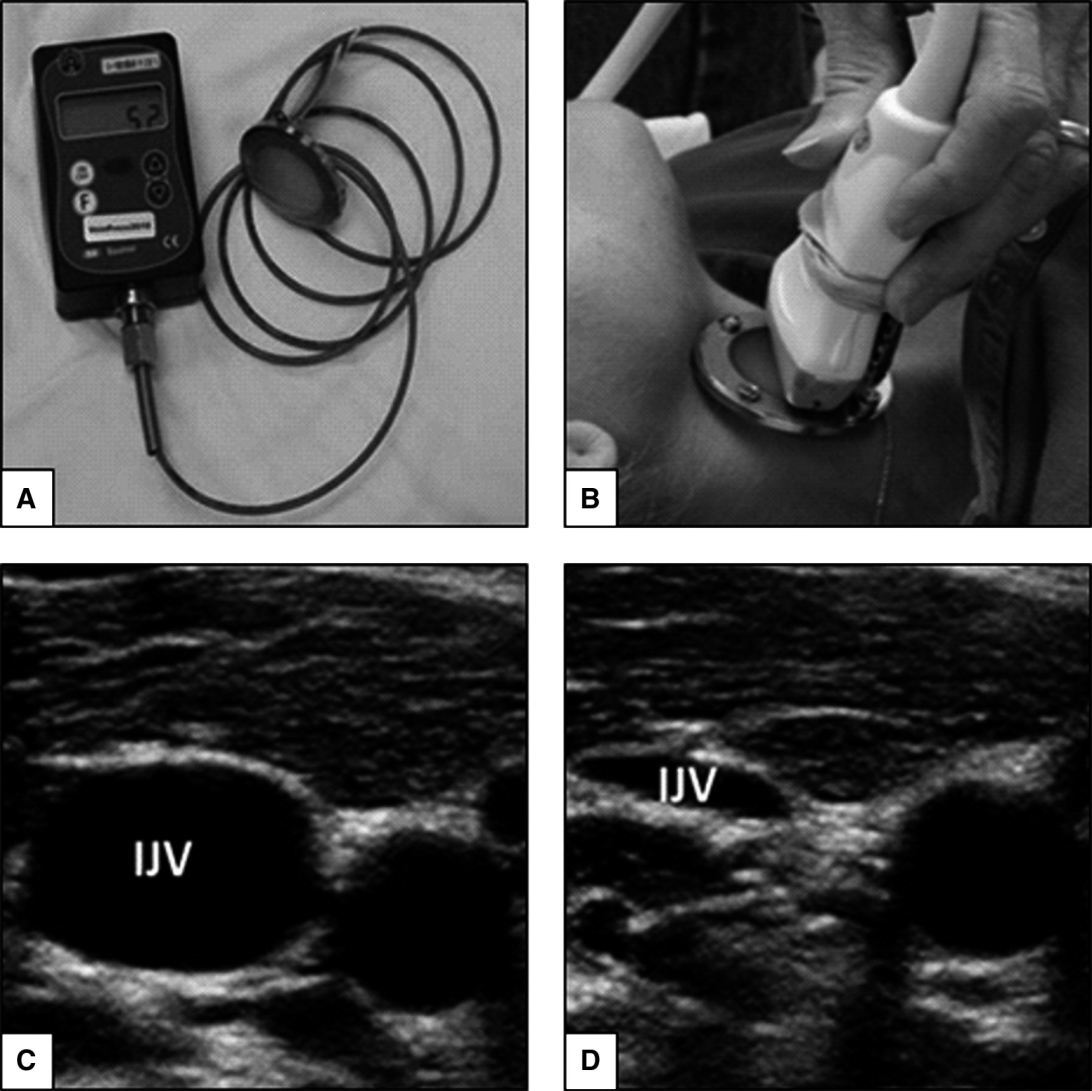
VeinPress device by itself (A) and positioned as it was used on the ultrasound probe during internal jugular venous pressure (IJVP) measurements (B). An image of an uncompressed (C) and mostly compressed (D) vein.

The VeinPress, a bladder filled with an ultrasound‐translucent mixture of water and glycerin, was connected to a manometer and attached to the head of a 12–5 MHz linear array ultrasound probe (GE Vivid Q, GE Healthcare, Milwaukee, WI) (Fig.**** 1). Prior to compression of the vein during 1G, the device was zeroed in accordance with the manufacturer's recommendations while floating the bladder in ultrasound gel just above the skin overlying the right internal jugular vein without compressing the skin. An experienced sonographer then slowly pressed the VeinPress against the skin overlying the internal jugular vein until the vein was compressed to the point of closure as verified by ultrasound imaging. In accordance with Laplace's law, the pressure that was necessary to compress the vein was assumed to equal the pressure within the vein. The pressure in the bladder was displayed digitally to an independent operator so that the sonographer was blinded to the pressure measurements. Once the operator recorded each measurement, the sonographer released the pressure for ~3 sec, with the VeinPress removed from contact with the skin, before repeating the measurement. Because there was insufficient time within each parabola to repeat the zeroing process, an alternative approach was employed during parabolic flight. The VeinPress was zeroed before each set of 10 parabolas. When the target gravity level was reached for each parabola the pressure displayed on the device was recorded while floating the bladder in ultrasound gel without compressing the skin. The sonographer then compressed the vein as described above for 1G measures, and the pressure displayed by the device at vein closure was recorded. The difference in the displayed pressure between the final pressure and the pressure reading without touching the skin represented the pressure required to compress the vein. Using this method, generally two measurements were acquired in each parabola. This procedure was repeated a minimum of 2–3 times per condition. Due to the respiratory dependency of IJVP, all measurements that did not involve breathing maneuvers were acquired at the end of a quiet exhalation.

Compression sonography measurements can be operator‐ and experience‐dependent. The sonographer performing the measurement must be able to clearly visualize the vein of interest, evenly apply pressure on the jugular vein using the probe, and record the pressure measurement at the point where the vessel walls just touch each other. The jugular vein is well suited for these measurements because of its size and distinct appearance of vessel walls imaged with sonography (Martin et al. 2015). In this study, only one sonographer experienced in compression sonography acquired all pre‐ and in‐flight data, thus limiting sources of error. Pressure readings and ultrasound images were recorded throughout the parabolic flights for later offline verification of the measurements obtained. Agreement between the replicate measures within subjects was excellent during 1G rest and 1G breathing maneuvers (ICC = 0.91–0.98), although somewhat less agreement was observed during 0G rest and 0G breathing maneuvers (ICC = 0.64–0.76). Lower agreement during 0G might be related to the dynamic nature of parabolic flight and the short window in which to acquire these data within a parabola.

### Data analysis and statistics

Three to five measurements of IJVP were obtained before parabolic flight and during 0G or partial gravity. Data were averaged within a subject and within a condition prior to statistical analyses. Differences in IJVP between 1G and 0G were tested using a paired T‐test. Respiratory maneuvers were performed during the 0G parabolas in the 7 of the 11 subjects. A mixed‐effects linear regression model was used to examine the effect of gravity and breathing maneuvers on IJVP using dummy‐coded grouping variables for gravity and pressure relative to 1G seated. Bootstrap resampling was performed to improve estimates of variance given the small sample size. We used the Holm correction for multiple comparisons between baseline and each treatment condition. Summary data are expressed as mean ± SD unless otherwise stated. All statistical analyses were performed using Stata/IC software (v13.1, StataCorp LP, College Station, TX, 2013) and setting two‐tailed alpha to reject the null hypothesis at 0.05.

## Results

**Figure 2:**
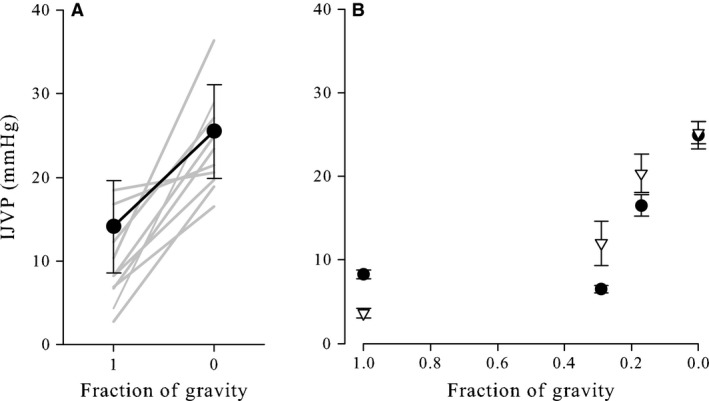
(Panel A) Mean (±standard deviation) internal jugular venous pressure (IJVP) was significantly lower (*P* ≤ 0.001) when supine before flight (Earth gravity, 1G) than during parabolic flight‐induced periods of weightlessness (0G). Individual results (*n* = 11) are displayed as gray lines. (Panel B) IJVP appeared to increase as gravitational load decreased from 1 g to 0G in two subjects during parabolic flight. Data from individual subjects are represented as mean ± standard deviation of 3–5 IJVP measurements.

Internal jugular venous pressure was significantly greater in 0G than 1G (23.9 ± 5.6 vs. 9.9 ± 5.1 mmHg, *P* < 0.001; Fig.**** 2). Although statistical analysis was not possible, it appears that IJVP increased as gravity levels decreased. In two subjects, IJVP did not increase consistently from 1G to Martian gravity (0.29G), but IJVP was greater during Lunar gravity (0.17G) and increased further in 0G.

**Figure 3:**
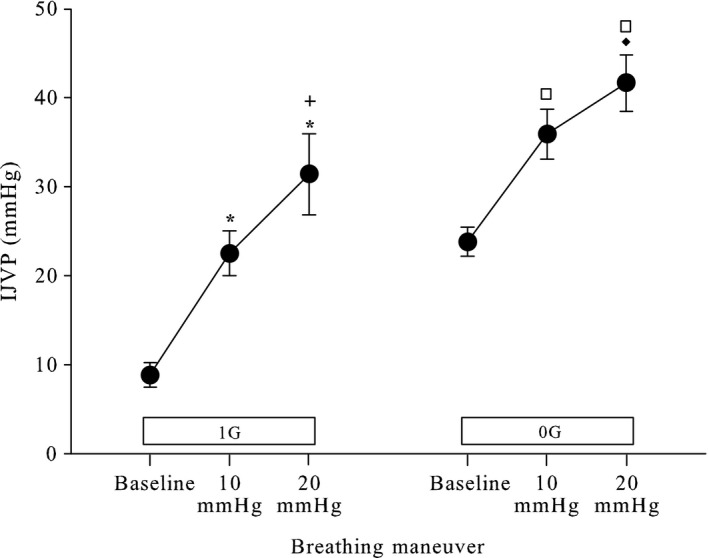
Mean (± standard deviation) internal jugular venous pressure (IJVP) assessed in 1G and 0G while conducting breathing maneuvers with expiratory pressures of 10 and 20 mmHg. 1G expiratory pressures elicited an increase in noninvasively measured IJVP that roughly approximated the change in intrathoracic pressure created by breathing maneuvers. (* <0.001 vs. 1G baseline, **+** =0.088 vs. 1G 10 mmHg, ♦ =0.066 vs. 0G 10 mmHg, □ <0.001 vs. 0G baseline).

The increase in IJVP measured during controlled Valsalva maneuvers roughly approximated the increase in expiratory pressures in 1G (Fig.**** 3). When subjects breathed out with an expiratory pressure of 10 mmHg, the IJVP increased 14 mmHg, (from 9.3 ± 4. 6 to 23.9 ± 7.1, *P* < 0.001) as compared to a normal tidal expiration. Breathing out with an expiratory pressure of 20 mmHg elicited an increase of 23 mmHg greater than baseline (*P* < 0.001) and also was 9 mmHg greater than the pressure measured at an expiratory pressure of 10 mmHg (23.9 ± 7.1 to 32.9 ± 12.9, *P* = 0.088). This pattern was similar when the controlled Valsalva maneuvers were performed in microgravity, although at higher pressures. In microgravity, when subjects breathed out at an expiratory pressure of 10 mmHg, IJVP increased by 12 mmHg (from 23.9 ± 5.6 to 36.2 ± 8.6, *P* < 0.001) from the microgravity baseline. Breathing out at an expiratory pressure of 20 mmHg elicited an IJVP 21 mmHg higher than the 0G baseline (36.2 ± 8.6 to 42.6 ± 8.4, *P* < 0.001). This IJVP represented an increase of 9 mmHg compared to the measurement at 10 mmHg of expiratory pressure (*P* = 0.066).

## Discussion

There were two major findings from this study. First, IJVP was greater during parabolic flight‐induced weightlessness than in 1G. IJVP measured during this acute exposure to 0G was approximately twice the IJVP observed in 1G while supine. Furthermore, although our data were limited to observations in two subjects, IJVP appears to increase as the level of gravity decreases. IJVP measured during parabolas simulating Martian and lunar gravity appeared to increase in a nonlinear fashion between 1G and 0G. Second, IJVP increased incrementally in 0G as intrathoracic pressure increased during the performance of controlled Valsalva maneuvers. That is, IJVP increased relative to normal expiration in both 1G and 0G in a fashion that corresponded to the increase in expiratory pressures. These results suggest that exposure to 0G or increased intrathoracic pressure induced by a Valsalva maneuver increases IJVP, which may be a contributing factor to the hypothesized increase in ICP in ISS astronauts. However, a relationship between changes in IJVP and the development of VIIP symptoms must be confirmed during long‐duration exposure to weightlessness.

The physiologic adaptations which contribute to the VIIP syndrome have not been clearly elucidated but the cephalad fluid shift that occurs upon entry in weightlessness has been hypothesized to be a significant contributor (Michael and Marshall‐Bowman 2015); approximately 2 L of fluid shift into the upper body from the legs during weightlessness (Thornton et al. 1987). Although a large portion of the fluid is eliminated from the vascular space, as evidenced by plasma and blood volume loss (Leach et al. 1996), some fluid continues to reside in intracellular spaces and in the veins of the upper body. During the 84‐day Skylab four mission, the jugular veins and veins of the head were reported to be completely full and distended, measured using infrared photography (Gibson 1977). Arbeille et al. (2001) also reported increased jugular vein size during stays on the Russian Mir space station. Anecdotally, distention of the internal jugular vein has persisted for the duration of ISS missions of 6 months. While our data were acquired during the brief periods of 0G during parabolic flight, long‐term engorgement of the internal jugular vein appears to persist beyond the initial entry into microgravity (Arbeille et al. 1999, 2001, 2015). A distended or engorged internal jugular vein might result either from an increase in venous pressure or a decrease in the pressure from surrounding tissue. For example, reduced external compression due to unweighting of the tissues overlying the chest cavity has been suggested to explain decreased CVP and increased left ventricular end‐diastolic volume observed during space flight (Buckey et al. 1996; Foldager et al. 1996). In contrast, there is little tissue overlying the internal jugular vein, which was chosen for this experiment because of its contributions to cranial drainage and its superficial location. During these parabolic flights we observed elevated IJVP when the vein was distended.

**Figure 4:**
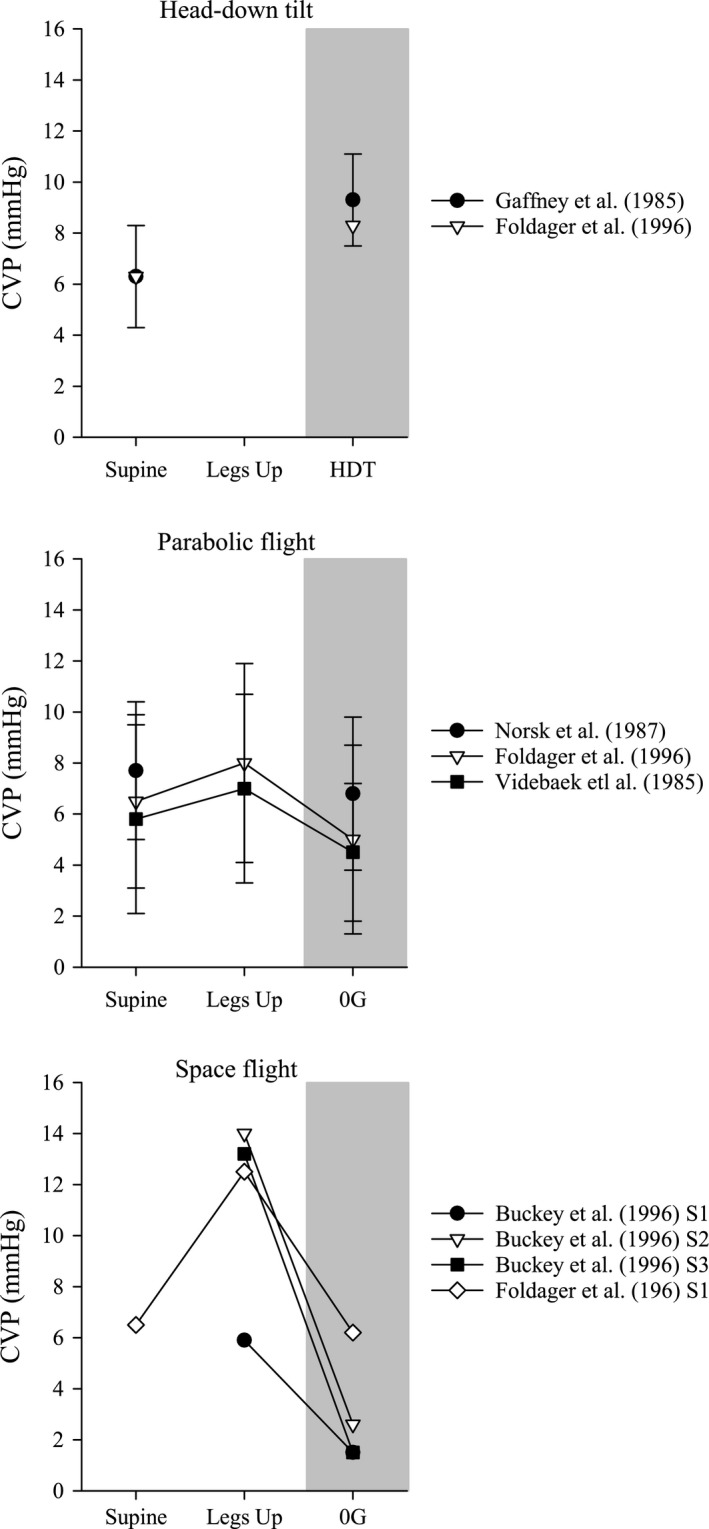
Direct measures of central venous pressure (CVP) while supine or supine with the legs elevated to simulate the prelaunch position on the Space Shuttle and during head‐down tilt (HDT), parabolic flight (0 g), or spaceflight (0 g). Data are redrawn from previously published results by Buckey et al. (1996), Foldager et al. (1996), Norsk et al. (1987), and Videbaek and Norsk (1997). Data are represented as mean ± standard deviation, except for individual results shown by subject in astronauts and during one subject in head‐down tilt Foldager et al. (1996).

**Figure 5:**
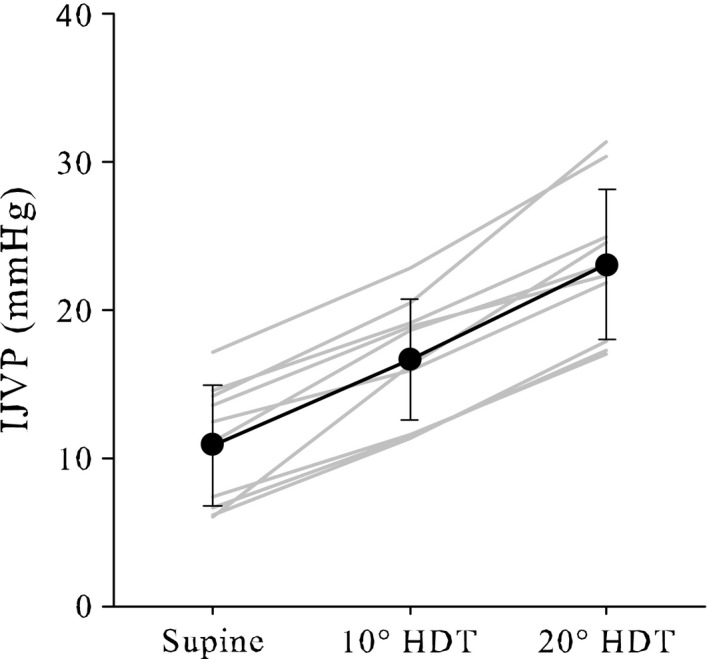
Mean (± standard deviation) internal jugular venous pressure (IJVP) measured in normal, healthy subjects while supine and during 10° and 20° head‐down tilt. Individual results (*n* = 10) are displayed as gray lines. Redrawn from Martin et al. (2015).

While seated upright or standing in 1G, venous flow from the head and neck is assisted by gravity and flow is unimpeded in healthy subjects. While in many individuals the jugular veins are flaccid when upright in 1G, distension of the jugular veins during simulated and actual weightlessness is a common observation (Arbeille et al. 1999, 2001, 2015). Head‐down tilt, one analog of spaceflight, produces distension of the jugular veins that likely results from a combination of a reversal of the hydrostatic gradient relative to the upright posture and an elevated CVP (Gaffney et al. 1985; Foldager et al. 1996) (Fig.**** 4). In our experience, jugular vein distension is coupled with an incrementally increasing IJVP as the head‐down tilt angle increases (Martin et al. 2015). Specifically, we observed that IJVP measured using compression sonography, using the same techniques as described here, increased by ~50% when moving from supine to 10° head‐down tilt, and IJVP at 20° head‐down tilt was almost twice as much the supine measurement (Fig.**** 5).

Interestingly, IJVP also increases during parabolic flight to levels almost twice that measured while supine in 1G. This occurs despite a decrease in CVP during parabolic flight, as has been reported previously (Foldager et al. 1996; Videbaek and Norsk 1997), which might be expected to increase venous outflow from the head and neck, thus counteracting venous congestion. In fact, venous return appears to be elevated during parabolic flight, based upon observations of increased atrial and ventricular volumes, mitral inflow velocity (E wave), stroke volume, and cardiac output (Videbaek and Norsk 1997; Caiani et al. 2006; Norsk et al. 2006; Petersen et al. 2011). Despite this, we observed that IJVP was elevated during weightlessness and increased from the supine posture in 1G through decreasing levels of gravity during parabolic flight. The increased cardiac filling and increased IJVP during parabolic flight may be the result of elevated venous return from the lower body with no change or a decrease in venous flow from the upper body. Even during the brief duration of weightlessness during parabolic flight, significant volumes of blood are displaced from the legs and abdomen toward the upper body (Bailliart et al. 1998; Petersen et al. 2011). An acute increase in venous return from the lower body might impair venous outflow from the head and neck when cranial venous drainage would not benefit from the assistance of gravity during weightlessness.

Data acquisition during parabolic flight occurred while the subjects were in the upright seated posture to facilitate access to the jugular vein by the sonographer, and this may have somewhat exaggerated IJVP above that which might have occurred had the subjects been supine during the hypergravity phase of the parabola immediately preceding weightlessness. CVP (Pantalos et al. 1999) and stroke volume (Petersen et al. 2011) increase from 1G to 0G when the subjects are seated throughout the parabola, including the hypergravity phase, but CVP decreases and stroke volume does not change from 1G to 0G when the subjects are supine during the entire parabola. Presumably, the upward movement of abdominal organs and blood trapped in the abdomen when seated during hypergravity contributes to the increases in CVP and stroke volume during 0G, but this effect would be minimized when subjects are supine throughout the parabola. Additionally, Norsk et al. (1987) reported that the seated posture in parabolic flight results in an elevated CVP when comparing to the supine position in 1G, in contrast to a decreased CVP reported by others when measured while supine in both 1G and 0G. However, given the magnitude of the observed increases in IJVP, a twofold increase, we are confident that the pattern of the response would have persisted.

While these IJVP data did not directly assess ICP, our findings are consistent with previous reports of internal jugular vein distension during spaceflight (Arbeille et al. 2001, 2015). Based upon our observations and others, one might speculate that this clear increase in IJVP during acute weightlessness could inhibit venous flow, reducing cerebrospinal and lymphatic fluid drainage from the skull resulting in increases in ICP (Mader et al. 2011; Nusbaum et al. 2013). However, venous pressure measures were obtained only in the right internal jugular vein, and other veins draining the cranium were not similarly interrogated; thus conclusions regarding total venous pressure based upon these IJVP data should be cautiously evaluated. While the anterior‐posterior hydrostatic forces that are present in the head while supine in 1G favor flow through the internal jugular veins, the effect of the absence in 0G of these forces during weightlessness on venous flow patterns has not yet been described. The internal jugular veins are a major drainage pathway between the brain and the heart, although the percentage of blood draining through the internal jugular vein in relation to the vertebral veins is position dependent (Manuel Valdueza et al. 2000; Cirovic et al. 2003). Along with an increased IJVP and venous distension, intraocular pressure also is elevated with parabolic flight (Mader et al. 1993; Anderson et al. 2016). While this study only reports a finding during acute exposure to weightlessness, increased IJVP during long‐duration spaceflight may promote cranial venous and lymphatic congestion chronically. Recent data suggest that ICP during parabolic flight is not different than that measured while supine in 1G (Lawley et al. 2015), but at this time no IJVP, IOP, and ICP measurements during space flight have been reported (Michael and Marshall‐Bowman 2015), and thus the relation between these and the development of the VIIP syndrome in long‐duration astronauts is yet to be established. To date, only postspace flight lumbar opening pressures have been reported in six astronauts who have demonstrated VIIP symptoms, and these were only acquired 7–66 day after landing (Mader et al. 2011; Lee et al. 2016). Current work in our laboratory seeks to describe the relation between fluid shifts, cardiovascular responses (including IJVP), noninvasive measures of ICP, and ocular function and structures during space flight in astronauts who do and do not develop VIIP symptoms.

The first reports of the VIIP syndrome in astronauts participating in long‐duration spaceflight appear to have coincided with the deployment of the ARED on ISS. The ARED allows for much higher loading during exercise countermeasures than available with previous exercise devices (Loehr et al. 2011), and thus ARED exercise has been suggested as a potential contributor to the VIIP syndrome. Valsalva maneuvers are commonly performed during resistive exercise and have been shown to transiently increase CVP (Wendling et al. 1994) and ICP (Haykowsky et al. 2003; Prabhakar et al. 2007). Thus, we sought to determine whether controlled Valsalva maneuvers would elevate IJVP above resting levels during weightlessness; we suspect that astronauts likely experience spikes in IJVP during resistive exercise countermeasures. Valsalva maneuvers were performed both to define the response of noninvasive IJVP measurement to a graded increase in intrathoracic pressure and to provide insight into the potential increase in IJVP during resistive exercise, if performed with a closed or semiclosed glottis. Based upon our observations of IJVP during mild Valsalva maneuvers, we would expect IJVP to increase dramatically during resistive exercise in spaceflight, albeit transiently. The link between ARED exercise and development of VIIP has not been proven, and it remains unclear whether transient elevations in IJVP, and presumably ICP, cumulatively across a long‐duration mission result in the development of VIIP.

### Limitations of the study

We have identified several limitations of the study design that might have impacted the results of this study and the applicability of our findings. First, the investigators recognize that despite previous validation of the technique in 1G and the controls implemented for the acquisition of IJVP during these acute 0G exposures, confidence in these findings would be enhanced if this noninvasive measure of IJVP using compression sonography had been validated against an invasive measure during weightlessness. Second, the pressures measured using compression sonography, even in 1G, may overestimate the actual pressures in the vein. That is, the character of the tissue overlying and underneath the vein of interest will impact how well the pressure from the transducer is transmitted to the vein. For example, stiff tissue over the vein will increase the amount of pressure required to compress it. While the absolute pressure measurements reported here might be elevated above the true IJVP, we consistently observed within‐subject trends for increasing pressures with weightlessness and breathing maneuvers. Furthermore, individual differences in tissue quality may limit the comparison of absolute values between subjects. Third, the lack of venous filling of the internal jugular vein in the upright position in 1G precluded a complete understanding across the spectrum from 1G seated to 0G. Although there was a clear increase in IJVP with decreasing gravity compared to the supine 1G condition, we were unable to make comparisons between IJVP while upright, postures in which individuals in 1G spend the majority of their day, and IJVP during weightlessness and partial gravity. Fourth, preflight data were collected approximately 1–2 h prior to flight and before the administration of an antiemetic medication (Scopolamine, ~45 min prior to flight). While oral scopolamine is associated with orthostatic hypotension in some individuals (Nuotto 1983), no subjects in this study reported any symptoms. Finally, application of these findings to and interpretation of measurements from astronauts participating in long‐duration space flight may be affected by adaptations to weightlessness, including plasma volume loss and changes in fluid compartmentalization (Leach et al. 1996) as well as changes in the tissue overlying the internal jugular vein. For example, while the stiffness of these tissues likely would not be expected to change during parabolic flight, neck muscle atrophy and fluid shifts from the intra‐ to extravascular spaces may impact the quality of the measurements during prolonged weightlessness.

## Conclusions

IJVP increases significantly during fluid shifts induced by parabolic flight intended to produce brief periods of weightlessness. Further, IJVP is elevated transiently by increased intrathoracic pressure induced by controlled Valsalva maneuvers in 1G and 0G. Especially given that the etiology of VIIP has not yet been clearly elucidated, future investigations are required to determine whether elevated IJVP persists, is indicative of venous congestion secondary to the cephalad fluid shift, and contributes to ocular disturbances during long‐duration spaceflight. Additional work also may reveal whether brief elevations in IJVP during the performance of exercise countermeasures in weightlessness also may contribute to the development of VIIP in astronauts. The capability to observe changes in IJVP noninvasively during long‐duration spaceflight using compression sonography could provide an ideal alternative to invasively measuring venous pressure over the course of long‐duration spaceflight and would contribute to NASA's endeavor to further understand the factors leading to the development of VIIP.
